# The Liver

**Published:** 1871-09

**Authors:** 


### THE LIVER

Is the largest "gland" or workshop of the body. Its office is to separate the "bile" from the blood, and pass it out of the system. This bile is waste matter which, having subserved its purpose, is not only no longer of use to the system, but if allowed to remain in it, poisons the blood, and makes it unfit for the purposes of life. The effects of this poisoning are varied, according to the part of the body most affected by it. In the head it causes headache, a dull, heavy feeling; the mind cannot readily concentrate itself on any subject; the memory is affected; the temper becomes irritable, morose, sour; low spirits, dizziness, and nervousness, with all the attendant miseries, make up the picture. In other cases, the skin takes on a diseased action, and there are fever sores on the lips, pimples on the face, discolored blotches on the forehead or other parts, boils, carbuncles, sores, and tumors. In other cases, the stomach becomes dyspeptic, the bowels costive, and nausea, loss of appetite, and general "miserableness" help to weary and worry a man's life out of him, ending in piles, dropsies, diarrhœas, and other debilitating forms of disease. All these result from the liver not working; when it is said to be "torpid," it is called a "lazy liver."

Persons who do not perspire much, suffer most from a torpid liver; and the man who eats his bread by the sweat of his brow — works for a living — has the best chance for a healthy and enjoyable life. Hence the remedy for a large portion of ordinary diseases is good honest daily labor. There are artificial methods of making the liver work, and the physician is called upon to practice these methods. In doing this, he gives medicines which are said to "act upon the liver," or, in vulgar phrase, to "stir up the bile." Various medicines do this; calomel is the easiest to take, is the most certain and effective in its action, and so is universally employed by the educated practitioner. Other medicines are "good for" the liver, such as the extract of the bark of the white walnut-tree, the extract of the tomato, and others, but all these lose their virtue by age, and at most are uncertain and unequal in their action; hence the physician, to make sure of it, and wishing to remove his patient's symptoms in the shortest time, and most certainly, resorts to calomel.

The connection between a torpid liver and torpid bowels, which means constipation, is this: the liver forms the bile and conveys it into the gall bladder, which empties itself into the bowels just under the stomach, or at their top or beginning, mainly during digestion, or while the food is passing out of the stomach. This bile "stimulates" the intestines, causing them to keep in motion, which motion passes the food onwards and downwards along the whole thirty feet in length of the intestinal canal, giving out its nourishment as it passes, until it comes to the bottom, when there is nothing left but refuse matter, to be passed out by the daily evacuation.

It is worth while to notice here the beautiful economy of means to an end, of the " Maker of our frame." This very bile, the refuse of the blood, is made to perform a most essential part of the bodily functions; for instead of making some substance for the express purpose of keeping the bowels in motion, in a healthy condition, the waste, useless, poisonous bile is made to perform that office.

But the great practical fact which the intelligent reader wishes to know is, How am I to keep the liver in healthful action? How am I to regulate the daily functions of the body, and have its health under my own control, without medicine and without work? There is one safe, efficient, and very certain method: spend a large portion of daylight in useful activities in the open air; at least walk several hours every day with some interesting object in view; for example, fall in love with some pretty girl or blooming widow, who lives five miles away, without any method of getting there except "footing it;" love her so hard that you feel obliged to see her once in every twenty-four hours; and if you don't get well of the worst liver complaint any man ever had, in a reasonably short time, it will be because physiology is a humbug, or you are not worth being cured. The sober truth is, that interested activities in the open air for a large portion of daylight, will, if persevered in, cure half the diseases to which mankind are subject, if not too long deferred.

